# Collaborative multicenter trials in Latin America: challenges and opportunities in orthopedic and trauma surgery

**DOI:** 10.1590/1516-3180.2013.1313555

**Published:** 2013-06-01

**Authors:** Vinícius Ynoe de Moraes, João Carlos Belloti, Flávio Faloppa, Mohit Bhandari

**Affiliations:** I MD. Attending Physician, Division of Hand and Upper Limb Surgery, Department of Orthopedics and Traumatology, Escola Paulista de Medicina, Universidade Federal de São Paulo (EPM-Unifesp), São Paulo, Brazil.; II MD, PhD. Professor, Division of Hand and Upper Limb Surgery, Department of Orthopedics and Traumatology, Escola Paulista de Medicina, Universidade Federal de São Paulo (EPM-Unifesp), São Paulo, Brazil.; III MD, PhD. Full Professor, Division of Hand and Upper Limb Surgery, Department of Orthopedics and Traumatology, Escola Paulista de Medicina, Universidade Federal de São Paulo (EPM-Unifesp), São Paulo, Brazil.; IV MD, PhD. Professor, Division of Orthopedic Surgery, Center for Evidence-Based Orthopedics, McMaster University, Hamilton, Ontario, Canada,

**Keywords:** Multicenter study [publication type], Fractures, bone, Traumatology, Orthopedics, Latin America, Estudo multicêntrico, Fraturas ósseas, Traumatologia, Ortopedia, América Latina

## Abstract

**CONTEXT AND OBJECTIVE:**

Orthopedic research agendas should be considered from a worldwide perspective. Efforts should be planned as the means for obtaining evidence that is valid for health promotion with global outreach.

**DESIGN AND SETTING:**

Exploratory study conducted at Universidade Federal de São Paulo (Unifesp), São Paulo, Brazil, and McMaster University, Hamilton, Canada.

**METHODS:**

We identified and analyzed collaborative and multicenter research in Latin America, taking into account American and Canadian efforts as the reference points. We explored aspects of the data available from official sources and used data from traffic accidents as a model for discussing collaborative research in these countries.

**RESULTS:**

The evaluation showed that the proportion of collaborative and multicenter studies in our setting is small. A brief analysis showed that the death rate due to traffic accidents is very high. Thus, it seems clear to us that initiatives involving collaborative studies are important for defining and better understanding the patterns of injuries resulting from orthopedic trauma and the forms of treatment. Orthopedic research may be an important tool for bringing together orthopedic surgeons, researchers and medical societies for joint action.

**CONCLUSIONS:**

We have indicated some practical guidelines for initiatives in collaborative research and have proposed some solutions with a summarized plan of action for conducting evidence-based research involving orthopedic trauma.

## RESUMO

**CONTEXTO E OBJETIVOS:**

A programação de pesquisa em ortopedia deve ser considerada do ponto de vista global. Esforços devem ser planejados como forma de se obter evidência que seja válida para promoção da saúde de alcance mundial.

**TIPO DE ESTUDO E LOCAL:**

Estudo exploratório conduzido na Universidade Federal de São Paulo (Unifesp), São Paulo, Brasil e na McMaster University, Hamilton, Canadá.

**MÉTODOS:**

Identificamos e analisamos pesquisas multicênticas/colaborativas realizadas na América Latina, considerando os esforços nos Estados Unidos e Canadá como referência. Para tal, exploramos os aspectos dos dados disponíveis em fontes oficiais e utilizamos os dados provenientes de acidentes de trânsito como modelo de discussão de pesquisas colaborativas nestes países.

**RESULTADOS:**

A avaliação demonstra uma pequena proporção de estudos colaborativos/multicêntricos em nosso meio. Análise breve demonstrou que existe enorme proporção de mortes devidas a traumas provenientes dos acidentes de trânsito. Dessa forma, parece-nos claro que iniciativas envolvendo estudos colaborativos são importantes para a definição e melhor entendimento do padrão das lesões provenientes de trauma ortopédico e as formas de tratamento. A pesquisa ortopédica pode ser importante ferramenta para aglutinar cirurgiões ortopédicos, pesquisadores e sociedades médicas para uma ação em conjunto.

**CONCLUSÕES:**

Apontamos algumas diretrizes práticas para iniciativas em pequisas colaborativas e propusemos algumas soluções com um plano de ação resumido para a realização de pesquisa baseada em evidências envolvendo trauma ortopédico.

## INTRODUCTION

Research attitudes should consider a worldwide scenario and be guided to improve patient health outcomes.^1^^,^^2^ Orthopedic surgery should also follow this approach. Illness due to orthopedic trauma is increasing in low and middle-income countries and should not be underestimated, since recent reports call this an “injury epidemic” that is far from being under control.^3^^,^^4^

As economic status strengthens in developing countries, individual incomes increase. This can lead to development of a unbalanced situation involving greatly increased use of motorized vehicles coupled with a lack of infrastructure and adequate road traffic policies to support this increase.^2^ This has led to increasing rates of disability and deaths due to road traffic trauma.^3^^,^^4^

Data summarized in the United Nations-supported First Global Ministerial Conference on Road Safety, which was held in Moscow, depict road traffic as a major cause of death for people aged between 5 and 29 years. It is well known that 90% of these deaths occur in low or middle-income countries.^3^^,^^4^ For Latin America, these data are sound. Trauma is a leading cause of death among boys and girls aged 5-14 years, as well as among economically active adult men and women.^5^

Orthopedic fracture demographics possibly follow this disturbing situation because environmental and lifestyle modifications may lead to injury patterns of greater severity. Unfortunately, no data from the orthopedic community on such injuries is available.^6^

This scenario suggests that collaborative multicenter studies would probably be the best tool that could be used to promote worldwide comprehensive research efforts within the orthopedic trauma setting. Such a research initiative would probably be the most effective tool for promoting evidence on a worldwide basis.^7^ It would be comprehensive because the trauma burden for different geographical areas would be included.^2^ Also, it would be strong with regard to the external validity of the results because of planning for wide-reaching promotion of orthopedic evidence-based research.

Examples of success in multi-collaborative research are widespread in non-orthopedic research.^8^^,^^9^^,^^10^ A comparative analysis conducted on studies published in 2009 demonstrated that up to 40% of the research published in highly-cited clinical journals, such as the New England Journal Of Medicine, Journal of the American Medical Association and Lancet, is produced through collaborative efforts.^11^

Clinical studies are more prone to have a higher number of institutions involved, and this has led to inclusion of higher number of participants in research reports. It is also true that straight comparisons in clinical research are not always reasonable, since some of them are produced through large drug industry-sponsored trials and are part of the unavoidable process for achieving clearance from the United States Food and Drug Administration (FDA).^11^ In this respect, the orthopedic research scenario is similar to other surgical specialties, such as gynecology and obstetrics and ophthalmology.^11^

## OBJECTIVES

The aim of this study was twofold: 1) to explore data from the published literature and official records from trusted organizations, pointing out the potential for conducting collaborative multicenter trials in Latin America; and 2) to discuss and present a rationale for research actions to be undertaken in Latin America.

## METHODS

### Data exploration: collaborative research as an opportunity to generate research in Latin America

For this exploratory analysis, we conducted a non-pragmatic Medline-based search strategy with the aim of finding collaborative research data. We conducted a search for randomized controlled trials within orthopedics and trauma (O&T) surgery, while focusing on two geographically and economically distinct groups: 1) Latin American countries; and 2) North American countries.

## RESULTS

**Figure 1.**
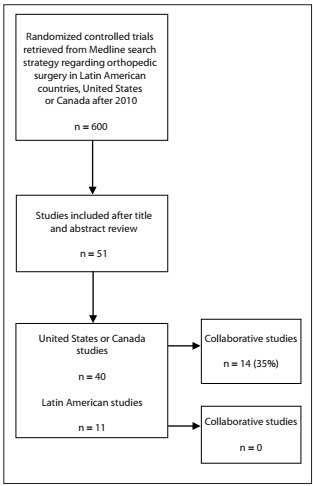
Data exploration assessment.

In Table 1, we show the search strategy used and the studies retrieved. By analyzing the titles and abstracts, we also identified the studies reporting collaborative or multicenter research (Table 1 and Figure 1).

**Table 1.** Search strategy and orthopedic studies retrieved*

Search strategy	Studies retrieved	Orthopedics - after analysis	Collaborative studies
1. ((Orthopedic Surgery) OR orthopedic surgery [MeSH Terms]) OR fracture fixation [MeSH Terms]	600		
2. Brazil OR Venezuela OR Argentina OR Colombia OR Peru OR Mexico	1276		
3. United States of America OR Canada	8110		
4. 1 AND 2	11	7	0
5. 1 AND 3	40	26	14

Limits: 2010-2012 (May), PubMed filter for randomized controlled trials.

These data show that there was a discrepancy in conducting high-level orthopedic-focused randomized trials, over a 2.5-year period, thus leading to an approximately fourfold difference in published randomized trials over that period. This is certainly not precise data, but it demonstrates the lack of high-quality research currently available.

**Figure 2.**
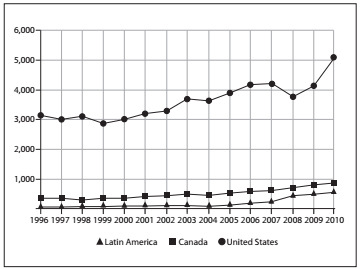
Plot from published studies filtered for Orthopedics and Sport Medicine: SJR SCImago Journal & Country Rank (SCOPUS database).

**Figure 3.**
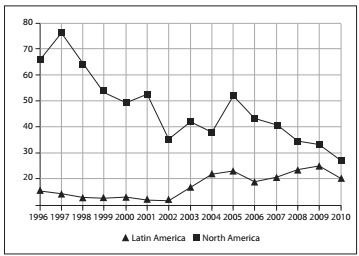
Plot depicting the percentage of collaborative studies filtered for Orthopedics and Sports Medicine: SJR SCImago Journal & Country Rank (SCOPUS database).

Data gathered from the SCOPUS database are also sound (www.scimagojr.com). These show that there are discrepancies in the rates of published articles from 1996 to the present day and in the proportion of collaborative studies (not only randomized trials), for orthopedics and sports medicine (Figures 2 and 3).

These comparisons were made considering United States, Canadian and Latin American data. Large volumes of research have been published in the United States, with a growth trend that is greater than the Latin American or the Canadian trend. With regard to the percentage of collaborative studies, the interpretation might be misleading for United States data, since the high absolute number of published papers might be responsible for the proportional drop in participation in collaborative studies.

Economic and demographic characteristics should be taken into consideration in analyzing these results. For example, the data in Table 2 shows that there is some equivalence of the populations for Latin America and North America, while there is a six to tenfold disproportion in country incomes. These points probably jeopardize some of the research initiatives, such as funding barriers and lack of infrastructure.

**Table 2.** Rates of fatal and non-fatal injuries in the Americas^3^^,^^4^

Country	Population	Gross national income per capita(US$)	Rate of RTF (10^3^ scale)	Rate of non-fatal injuries (10^3^ scale)	Trends in road traffic deaths
United States	305, 826,246	46,040	0.14	10.81	Decreasing
Brazil	191,790,929	5,910	0.18	2.13	Stable
Mexico	106,534,880	8,340	0.16	5.67	Stable
Colombia	46,155,958	3,250	0.12	0.84	Decreasing
Argentina	39,531,115	6,050	0.10	4.41	Increasing
Canada	32,876,047	39,420	0.09	6.06	Decreasing

RTF = road traffic accidents.

Some good-quality Latin American research might be underreported or might be unreachable through our methodology. Nonetheless, systematic assessments for Latin American databases have shown that no extensive good-quality orthopedic research was published even after 2000.^12^^,^^13^

### The burden of road traffic accidents: a model for discussing the importance of orthopedics and traumatology research

The total Latin American population is around twice the populations of the United States and Canada combined. Developing countries such as Brazil, Mexico, Colombia and Argentina are probably experiencing the same injury trends that are reported in India and China.^3^^,^^4^^,^^14^

Injuries due to road traffic accidents (which include motorcycle, car and pedestrian accidents) might be underreported, since non-fatal injuries are more prone to be underestimated in lower income countries.^15^ Despite the well-known explicit nature of unrefined data, little is known about the real picture of orthopedic trauma injuries in Latin America (such as fracture patterns and treatment facilities and types), given that no comprehensive observational report is available. The orthopedic research community should focus some effort on this area.

**Figure 4.**
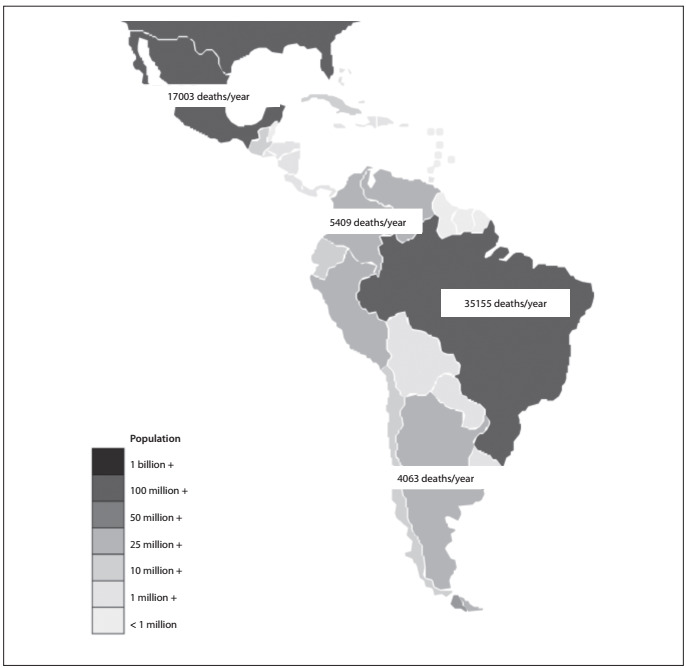
Map of Latin America showing population and absolute numbers of road traffic injuries according to legend bar depicting country populations. Absolute numbers of road-traffic fatalities are superimposed on each country that was assessed regarding road traffic accidents, from top to bottom: Mexico, Colombia, Brazil and Argentina. Data gathered from World Health Organization reports.^3^

In Figure 4 and Table 2, we demonstrate the importance of incorporating Latin American data across all research disciplines by taking the ubiquitous example of road traffic accidents. Latin America contains highly populated countries with high proportional rates of road traffic deaths and impairment.

## DISCUSSION

### A call for action for Latin America: comments

The proposal to carry out orthopedic research as a collaborative endeavor is challenging, and this has been well stated in some comprehensive discussions on this issue.^16^^,^^17^ Three years ago, a symposium supported by the American Academy of Orthopedic Surgeons (AAOS), the Orthopedic Research and Education Foundation (OREF) and the National Institute of Arthritis and Musculoskeletal and Skin Diseases (NIAMS) took this worldwide endeavor into account with regard to evidence.^17^

Different authors have pointed out what they judge to be the key factors that can lead to limitations or failure of a clinical trial project. These authors have identified the following relevant factors: cultural issues, investigator skills, academic barriers, infrastructure deficits, data management, institutional review board issues, trial regulation and funding.^17^

Although collaborative studies are more likely to be planned as randomized controlled trials, researchers should also consider assessing orthopedic issues through observational studies.^18^ In particular, observational studies could prove very useful for exploring research questions that are important today, and for gathering epidemiological data for public health initiatives.^18^ In Table 3, we have tried to summarize a rationale for action in Latin America.

**Table 3.** Steps for initiating a collaborative multicenter trial

Steps	Actions and possible solutions
1. Search and appraise	Perform a comprehensive search for the available evidence; contact researchers in the area to avoid duplication of efforts; appraise the quality of the existing evidence; and plot data for future steps, including probable difficulties that researchers might face in the study methodology and barriers to practical conduct of the studies.
2. Bring together and discuss	Provide leadership meetings and emphasize the need for and importance of collaborative research projects. Engage and recruit researchers from various orthopedics and traumatology societies. Explore the key health questions or areas of study, and discuss plans for research rationale and methodology. Searching for funding sources and strategizing for securing them are the cornerstones in this phase.
3. Motivate and conduct	Coordinating centers should maintain ongoing and regular communication with collaborative centers by requesting regular progress reports. Reports should include rates of patient inclusion, completeness of subject follow-up, as well as losses to follow-up and unanticipated challenges or complications. Experienced research coordinators are important factor for ensuring the feasibility and completion of long-term research projects.
4. Report and export	Data collection and extraction should be dynamic: reporting the results for a worldwide audience, ratifying the various research skills, and rewarding the research team.
